# An investigation of diet quality across racial groups in the United Kingdom and United States considering nutritional adequacy, disease risk, and environmental sustainability: a secondary analysis of NDNS and NHANES datasets

**DOI:** 10.1017/jns.2024.64

**Published:** 2024-12-13

**Authors:** Grace Bennett, Eileen R. Gibney

**Affiliations:** 1 UCD Institute of Food and Health, University College Dublin, Dublin 4, Ireland; 2 School of Agriculture and Food Science, University College Dublin, Dublin 4, Ireland

**Keywords:** Diet quality, Population health, Racial groups, Sustainability, AHEI-2010, Alternative Healthy Eating Index 2010, DQI-I, Diet Quality Index International, HEI, Healthy Eating Index, NDNS, National Diet and Nutrition Survey, NHANES, National Health and Nutrition Examination Survey, SSB, Sugar sweetened beverages, U.K., United Kingdom, U.S., United States, USDA, United States Department of Agriculture

## Abstract

Diet indices are quantitative assessments of the quality of population intake. Understanding diet quality is crucial to support health and well-being; however, knowledge of diet quality across racial groups is limited. To examine diet quality of acial groups ‘White’, ‘Black’, ‘Asian’, and ‘Other’ in the United Kingdom (U.K.) and United States (U.S.), U.K. and U.S. national survey data were used to calculate Alternative Healthy Eating Index (AHEI-2010), Diet Quality Index-International (DQI-I), and EAT-Lancet scores. ANCOVA tests compared median total quality scores across racial groups adjusting for covariates. Kruskal–Wallis tests examined differences in individual component scores. Spearman correlations identified association of diet quality scores across indices. Highest diet quality scores were reported for U.K. and U.S. Asian groups. Most noticeable differences were apparent between U.S. Asian and White/Black groups (62% Asians within highest tertile of AHEI-2010 score vs. 29% Whites; P < 0.001). All racial groups demonstrated poor diet quality in terms of sustainability; EAT-Lancet scores were <40% of maximum total score for U.S. White, Black, and Other groups. AHEI-2010 diet quality scores were moderately associated with EAT-Lancet scores, evident across all groups (r = 0.53–0.65; P < 0.001). There is a need for all groups to increase intake of wholegrains, especially Black groups (mean Wholegrain score for U.S. Black group within DQI-I was 0.60 (maximum score of 5)) as demonstrated within AHEI-2010, DQI-I, and EAT-Lancet component scores. Additionally, increased intake of vegetables and legumes and decreased intake of processed and red meat would improve the adequacy, healthiness, and sustainability of U.K. and U.S. racial diets.

## Introduction

Surveillance of dietary intake is essential to understand current consumption trends and indicate aspects of the diet which require more targeted guidance or intervention to maximise population nutrition and health.^(1–3)^ The diet can be assessed by examining individual food group or nutrient intake, or by considering the totality of intake, implementing diet quality indices to assess adequate consumption of dietary components critical to health and well-being.^(1,4,5)^

A consensus for what constitutes total diet quality is lacking and while it is deemed to be multifaceted, as far as the authors are aware, little research to date has assessed population diet quality considering multiple aspects collectively.^(6–8)^ Globally numerous diet quality indices have been developed to assess public adherence to a specific aspect of healthy eating recommendations, such as adequate nutrient intake, prevention of disease or sustainability of intake.^(2,9,10)^ The Alternative Healthy Eating Index (AHEI-2010), Diet Quality Index-International (DQI-I), and the EAT-Lancet Index are examples of dietary indices developed to examine the quality of these aspects of habitual diet individually (disease risk, adequacy, and sustainability, respectively).^(11–13)^ While each index has been developed for a specific purpose, there is scope to assess population intake using these indices in combination to identify total diet quality under the terms aforementioned. Detailing how such elements of diet quality rate and compare will inform how future dietary guidelines can support improved population consumption, encapsulating both adequate nutrition and optimum planetary health.

In addition to investigating diet quality in terms of the total diet, diet quality of the total population must also be understood. While many studies examine quality across age, sex or social groups, to date research has largely failed to analyse across racial or ethnic groups specifically.^(14–16)^ A recent systematic review identified just six studies globally that examined diet quality across racial/ethnic groups in one country (namely the United States (U.S.), The Netherlands, and Australia).^(14)^ In 2006, Nicolaou *et al.,* reported significantly poorer diet quality among Dutch respondents when compared to Surinamese ethnic groups, results supported by Yau *et al.,* in 2020.^(17,18)^ As well as identifying differences in diet quality across racial and ethnic groups this review also highlighted that many racial and ethnic groups are neglected in nutrition research, even within literature claiming to be *‘racially diverse’* (the U.S. studies identified only considered White and Black groups).^(14,19–21)^ As the developed world becomes increasingly diverse, it is essential current diet quality of racial groups within the population is identified to determine where targeted improvements to cultural diets are needed, informing public health policy inclusive of diverse groups within one locality.

To provide a comprehensive insight into total diet quality of diverse populations, the aim of this research was to determine multidimensional aspects of diet quality across racial groups within a country, considering if diet quality was comparable to other racial groups within that country and to the same racial groups living elsewhere. Two of the most diverse countries globally include the U.S. and the United Kingdom (U.K.), and as such, this paper utilised existing national survey data from the U.K. (the National Diet and Nutrition Survey (NDNS)) and the U.S. (National Health and Nutrition Examination Survey (NHANES)) to determine diet quality across racial groups.^(22,23)^ Although distinct aspects of the diet will be assessed independently using DQI-I, AHEI-I, and the EAT-Lancet index, the association between diet quality scores across indices will be examined, indicating whether a sustainable diet also adheres to dietary recommendations and is preventative of disease. Outcomes from this paper will identify intake of specific dietary components, that if changed, may improve the adequacy, healthiness, and sustainability of current diets across U.K. and U.S. racial groups.

## Methods

### Data collection

#### National diet and nutrition survey (U.K.)

Demographic and dietary intake data of adult participants (aged 18 years and above) from NDNS years 9–11 (2016–2019) were included in this secondary analysis.^(22)^ Within the NDNS study, demographic data were collected using structured questionnaires and 4-day food diaries were used to record dietary intake.^(22)^ Only participants who completed all four food diaries were included in this analysis. As part of the NDNS demographic questionnaire, participants classified their race as ‘White’, ‘Black’, ‘Asian’, and ‘Other, including mixed race’. The NDNS dataset provides intake of individual food and drink items and aggregates this data into food group intakes; these existing aggregated food groups were used to calculate food group components of the three diet quality indices (AHEI-2010, DQI-I, and EAT-Lancet). More detailed information about the NDNS can be found on the Public Health England website.^(22)^ All NDNS data included in this secondary analysis were freely available and downloaded directly from the U.K. data service website.^(22)^

#### National health and nutrition examination survey (U.S.)

Demographic and dietary intake data of adult participants (aged 18 years and above) from NHANES 2017–2018 were also included in this secondary analysis. Structured interviews were conducted to collect demographic data and dietary intake was assessed using 2-day 24-hour dietary recalls following the United States Department of Agriculture (USDA) Automated Multiple-Pass Method.^(23,24)^ Only participants who recorded 24-hour dietary recalls on both days were included in this analysis. During interviews participants classified themselves as ‘White Non-Hispanic’, ‘Black Non-Hispanic’, ‘Asian Non-Hispanic’, ‘Mexican American’, and ‘Other Hispanic’ and ‘Other including multi-racial’. In this secondary analysis, the groups ‘Mexican American’, ‘Other Hispanic’, and ‘Other including multi-racial’ were merged into a single racial group ‘Other’ to facilitate cross-country comparisons with the NDNS data.^(23)^ The USDA has converted intake of individual food and drink items reported in NHANES into food pattern components (Food Patterns Equivalent Intakes Database), and this database was used to calculate scores of food group components of each diet quality index.^(25)^ All data used in this secondary analysis were open access, and more information on NHANES is available on the National Center for Health Statistics website.^(23)^

This study was conducted according to the guidelines laid down in the Declaration of Helsinki, and all procedures involving human subjects/patients were approved by the National Health Service Health Research Authority Research Ethics Committee East of England Cambridgeshire South; approval number 13/EE/0016 (NDNS data) and the National Center for Health Statistics Research Ethics Committee; approval numbers #2011-17 and #2018-01 (NHANES data). Written informed consent was obtained from all subjects/patients.^(23,26)^ Ethical approval for this secondary analysis of NDNS and NHANES data was granted by the UCD Research Ethics Committee in May 2023 (S-LRSD-23-98-Bennett-Gibney).

### Selection of appropriate diet quality indices

#### Alternate healthy eating index-2010

The AHEI-2010 was developed as a proxy measure of disease risk attributable to habitual diet examining intake of food groups and nutrients particularly important to chronic disease aetiology.^(27–29)^ Although food-based dietary guidelines are designed to protect health, previous work has demonstrated associations between AHEI-2010 score and cardiovascular disease or cancer mortality, validating its use over other indices to assess diet-related disease risk.^(12,29)^ Inequalities in rates of modifiable diseases including cardiovascular disease are prevalent across population subgroups, where minority racial and ethnic groups report higher disease incidence and mortality.^(30–33)^ While changes to habitual dietary intake would likely mitigate these inequalities, cohort-specific dietary challenges need to be identified, and so the AHEI-2010 was implemented to assess dietary related disease risk among racial groups in the U.K. and U.S.

#### Diet quality index-international

To determine adherence to U.K. and U.S. dietary guidelines, which differ in their recommendations and targets, the Diet Quality Index-International was implemented. The DQI-I was developed to facilitate cross-country comparisons when assessing population adherence to dietary guidelines and nutritional adequacy of population intake.^(11)^ Different from many existing dietary indices, the DQI-I is flexible in terms of the conditions for component minimum-maximum scores allowing these scores to be based on country-specific guidelines, providing a cross-country comparison of adherence to relevant population food-based dietary guidelines and a standardised quantitative scoring approach (i.e. using the one diet quality index for U.K. and U.S. racial groups).

#### EAT-Lancet index

Assessment of dietary sustainability is critical to understand how food habits could be altered to mitigate the impact of food production, consumption, and waste on the environment.^(34–37)^ However not only should sustainable diets be *‘low in environmental impact…contributing to food and nutrition security and to healthy life for present and future generations’,* they need also be culturally acceptable, affordable, and accessible to all within the population.^(37)^ To safely improve the sustainability of food consumption, ensuring micronutrient sufficiency is not jeopardised with the transition to plant-rich diets, careful consideration across population subgroups is required.^(38)^ As such, the EAT-Lancet index created by Stubbendorf *et al.,*2022, which measures adherence to optimum sustainable diets based on key food components of EAT-Lancet recommendations, was applied to identify environmental sustainability of intake across U.K. and U.S. racial groups.^(13)^

### Calculating diet quality scores

Diet quality scores were considered in terms of disease risk (AHEI-2010), dietary adequacy (DQI-I) and environmental sustainability (EAT-Lancet). AHEI-2010, DQI, and EAT-Lancet scores were calculated for U.K. and U.S. cohorts separately. Based on original scoring criteria of AHEI-2010 and DQI-I, which accounted for different nutritional requirements by sex, AHEI-2010 and DQI-I component scores for the U.K. and U.S. were calculated for males and females separately, before combining male and female specific scores to calculate total population scores. EAT-Lancet did not provide sex-specific scoring criteria, and so EAT-Lancet scores were calculated for male and female participants jointly. A gradual scoring approach was applied to each component of the AHEI-2010, DQI-I, and EAT-Lancet index, to apply suitable scores to a tighter range of intakes; the scoring approach implemented for each diet quality index is available in Supplementary Table 1a–c. For example, when calculating AHEI-2010 component score, participants who consumed 4 or more servings of fruit daily (100% of recommendation) achieved the maximum score of 10, those who consumed 2 servings of fruit daily (50% of recommendation) achieved a score of 5, and those that had no servings of fruit scored 0; rather than participants receiving a score of 10 for meeting the recommendation and a score of 0 for consuming under 4 servings. For the purpose of this analysis, minor amendments to original AHEI-2010, DQI-I, and EAT-Lancet indices were made due to the availability of specific variables in NDNS and NHANES datasets, more detail of these amendments are described below and in Supplementary Table 2.

#### Alternate healthy eating index-2010

The original AHEI-2010 consists of 11 components (Fruit, Vegetables, Wholegrains, Sugar Sweetened Beverages & Fruit Juices, Nuts & Legumes, Red & Processed Meat, Trans-Fat, DHA & EPA, Polyunsaturated Fatty Acids, Alcohol, Sodium), all equally weighted at 10 points, meaning that total AHEI-2010 is scored out of 110 (favourable dietary pattern equal to higher AHEI-2010 score).^(12,39)^ However, due to the data variables available in NDNS and NHANES datasets some amendments were made to original AHEI-2010 scoring. These amendments are described in detail in Supplementary Table 2. Briefly, the variable ‘trans-fat’ (one of the 11 components of original AHEI-2010 scoring) was not available in NHANES and so this variable was not included, meaning that the AHEI-2010 scores in this analysis are out of a total of 100 (10 components scored at 10 points each). This approach has been adopted by others using NHANES data to calculate AHEI-2010 scores.^(28)^ When calculating AHEI-2010 scores for NDNS participants ‘trans-fat’ was also not used to ensure that AHEI-2010 scores for U.K. and U.S. populations were based on the same number of components. Additionally, within the NDNS cohort, some estimations were made to determine AHEI-2010 component scores of ‘DHA & EPA’, ‘wholegrain’, and ‘alcohol’ as this data was not readily available from the NDNS dataset (see Supplementary Table 2 for full explanation).

#### Diet quality index-international

The DQI-I is categorised into four sections; variety of food groups and protein sources (scored 0–20); adequacy of fruit, vegetables, wholegrains, fibre, protein, vitamin C, iron, and calcium intake based on national recommendations (scored 0–40); moderation of empty calorie foods, total fat, saturated fat, and cholesterol (scored 0–30); and balance of macronutrient and fatty acid intake (scored 0–10), equalling a total DQI-I score of 100, with a higher score indicating higher diet quality.

Interval cut-offs as previously described by Kim *et al.,* 2003 were applied to each component of the DQI-I for both NDNS and NHANES data.^(11)^ Within the DQI-I, adequacy of food group and nutrient intakes are based on country-specific guidelines and so *‘Government Dietary Recommendations’* for U.K. adults aged 19 years and above, published by Public Health England, were applied to NDNS data and USDA *‘Dietary Guidelines for Americans 2020–2025’* recommendations for adults aged 19–51 years were applied to NHANES data.^(40,41)^ Iron was the only nutrient to be calculated separately by age for both NDNS and NHANES data (already calculated separately by sex), allowing for age-specific iron recommendations for pre- and postmenstrual women. Intake of certain DQI-I components needed to be estimated as they did not explicitly exist in NDNS/NHANES datasets, these included ‘empty calorie foods’ (estimated for both NDNS and NHANES cohorts) and ‘wholegrain’ (estimated for NDNS cohort only). A detailed explanation into how this was done for each variable is available in Supplementary Table 2.

#### EAT-Lancet index

The EAT-Lancet index developed by Stubbendorf *et al.,* 2022 measures adherence of 14 dietary components to EAT-Lancet recommendations (Fruit, Vegetables, Wholegrains, Unsaturated oils, Legumes, Nuts, Fish, Beef and lamb, Pork, Poultry, Eggs, Dairy, Potatoes, and Added sugar), which are key for environmentally sustainable dietary intake.^(13,36)^ The EAT-Lancet index is scored 0–42, where all 14 components are equally weighted: a score of 3 (i.e. maximum score) indicates optimal adherence to EAT-Lancet recommendations.^(13,36)^ In this paper, all components were calculated as Stubbendorf *et al.,* 2022 describe except for the component ‘Beef and lamb’ where intake of all red meat including processed red meat were included in this component for both NDNS and NHANES datasets. In the NHANES dataset, all 14 components listed in the EAT-Lancet index were available, however food intake was reported as servings or ounces per day in the Food Patterns Equivalent Intakes database and so these were transformed into grams per day when calculating EAT-Lancet scores for NHANES participants.^(25)^ For NDNS data, intake of wholegrain, added sugar, and unsaturated oils was estimated to determine scores of ‘wholegrain’, ‘added sugar’, and ‘unsaturated oils’ components (see Supplementary Table 2 for more details).

### Analysing diet quality scores

AHEI-2010, DQI-I, and EAT-Lancet index scores were calculated for NDNS and NHANES cohorts on an individual level (as per approaches described above), and scores were then analysed using SPSS version 27 at a total population and racial group level by country.

Kruskal Wallis tests were performed to compare demographic characteristics across diet quality tertiles. To determine overall diet quality across U.K. and U.S. racial groups in terms of dietary adequacy; healthiness; and environmental sustainability, median total scores and interquartile ranges of AHEI-2010, DQI-I, and EAT-Lancet were calculated. Analysis of covariance (ANCOVA) examined the differences of total diet quality scores across racial groups (both within and across countries), controlling for covariates sex (NDNS)/gender (NHANES), age, education, and income levels, applying Bonferroni correction method. These covariates are widely considered in nutritional research as being influential to dietary behaviour and food intake and in this case allow examination of association between diet and race while controlling for the impact of these confounding factors.^(42–44)^ To understand what aspects of dietary intake account for variances in diet quality index scores across racial groups, scores of individual index component scores are presented across racial groups to permit more detailed investigations of dietary habits. Spearman correlations were performed to identify the relationship of scores across diet quality indices, determining if there is potential synergy among the aspects of diet quality considered. Statistical significance was considered at a 95% confidence interval.

## Results

### Participant demographics

An examination of demographics and socio-economic status across racial groups within the U.K. (NDNS dataset) and the U.S. (NHANES dataset) is available in Table 1. A total of n = 1780 U.K. and n = 4339 U.S. participants were included in this secondary analysis of diet quality: 90% of NDNS participants reported to be White while the NHANES cohort was more evenly distributed across racial groups (White = 36%, Black = 25%, Asian = 12%, Other = 27%, Table 1). In the U.K., the racial group Other consisted of *‘Mixed’*; n = 12 and *‘Any other group’*; n = 19. In the U.S., the racial group Other consisted of *‘Mexican American’*; n = 558, *‘Other Hispanic’*; n = 389 as racial categorisation of Hispanic/Latino participants were not asked in NHANES, and *‘Other Race – including multi-racial’*; n = 222. An even distribution across sex/gender groups was reported for all racial groups in both the U.K. and U.S. except for U.K. Black, where participants were mostly female (78%). In both countries, a BMI of ≥30 kg/m^2^ was most prevalent among White and Black groups and the U.S. Asian group had the highest proportion of respondents with a BMI between 18.5 and 24.9 kg/m^2^ (45% *vs*. ≤25% among non-Asian U.S. racial groups). A high percentage of non-White U.K. racial groups were educated to university level (47–60% *vs*. 32% of U.K. White) and there was an even proportion of U.K. racial groups across income levels. In the U.S., one third of the racial group Other reported having no qualification with <13% having a university degree, compared to 62% of the Asian group reporting the highest education level. The U.S. Asian group had the highest proportion (59%) of respondents with a household income of >$65,000 (highest income bracket). Demographic characteristics did differ across tertiles of diet quality scores including age, sex, education and income. In both U.K. and U.S. populations, tertile of AHEI-2010, DQI-I and EAT-Lancet scores differed significantly across racial groups (P ≤ 0.015); U.K. and U.S. Asian groups had the highest proportion of respondents whose scores fell within the highest tertile of AHEI-2010 and EAT-Lancet score (Supplementary Tables 3a–c).

**Table 1.** Participant demographics of NDNS and NHANES cohorts (U.K. vs. U.S. )

		United Kingdom (NDNS, n = 1780)				United States (NHANES, n = 4339)			
Variable		*White (n = 1614)*	*Black (n = 36)*	*Asian (n = 96)*	*Other (n = 31)*	*White (n = 1570)*	*Black (n = 1070)*	*Asian (n = 530)*	*Other (n = 1169)*
Age (yrs)	Mean (SD)	49.8 (17.9)	41.7 (13.6)	39.9 (13.5)	38.5 (13.0)	53.2 (19.5)	49.8 (17.4)	46.4 (16.4)	46.9 (30.4)
Sex; n (%)	Female	945 (58.6)	28 (77.8)	50 (52.1)	20 (64.5)	806 (51.3)	568 (53.1)	278 (52.5)	615 (52.6)
BMI; n (%)	18.5–24.9	555 (34.4)	10 (27.8)	32 (33.3)	13 (41.9)	394 (25.6)	222 (20.7)	236 (44.5)	204 (17.5)
	25.0–29.9	584 (36.2)	7 (19.4)	46 (47.9)	12 (38.7)	446 (28.4)	265 (24.8)	196 (37.0)	401 (34.3)
	≥30	429 (26.6)	13 (36.1)	13 (13.5)	6 (19.4)	685 (44.5)	544 (50.8)	83 (15.7)	529 (45.3)
Education; n (%)	No Qual*	632 (39.2)	14 (38.9)	35 (36.5)	6 (19.3)	179 (11.4)	151 (14.2)	46 (8.7)	389 (33.3)
	High School	367 (22.8)	5 (13.9)	11 (11.5)	7 (22.6)	1026 (65.4)	708 (65.7)	158 (29.8)	628 (53.8)
	Bachelors+	591 (31.9)	17 (47.3)	46 (48.0)	18 (60.2)	365 (23.2)	209 (19.5)	326 (61.5)	148 (12.7)
Income Level; n (%)	Low^	415 (25.7)	10 (27.8)	31 (32.3)	8 (25.8)	262 (17.4)	217 (20.3)	34 (6.4)	221 (18.9)
	Middle^	483 (29.9)	12 (33.3)	23 (24.0)	4 (12.9)	669 (44.4)	414 (38.7)	142 (26.8)	488 (41.7)
	High^	510 (31.6)	8 (22.2)	20 (20.8)	14 (45.2)	575 (38.2)	307 (28.7)	315 (59.4)	358 (30.6)

U.K., United Kingdom; U.S., United States of America; U.K. ‘Other” group includes mixed race, U.S. ‘Other” group includes Hispanic, Mexican American and multiracial groups, age measured in years, SD, standard deviation, BMI categories based on WHO classification; the proportion of participants with a BMI of <18.5 were not reported in this demographics table, No Qual* = no qualification, + = Bachelors degree and above, ^ = NDNS categorised income into three tertiles, NHANES reported incomes were categorised into these tertiles based on distribution (low <$20,000, middle =$20,000–$64,999, high = $65,000+).

**Table 2a.** AHEI-2010 component scores across racial groups (U.K. and U.S.)

United Kingdom (U.K.)									
*AHEI-2010 Components*	*White*		*Black*		*Asian*		*Other*		
Score Range; 0 (lowest)–10 (highest quality)	Mean (SD)	Percent	Mean (SD)	Percent	Mean (SD)	Percent	Mean (SD)	Percent	P value
Fruit	2.78 (2.87)	27.75	3.20 (3.62)	32.00	2.82 (8.83)	28.20	2.86 (2.72)	28.65	0.940
Vegetables	2.33 (2.21)	23.29	2.63 (2.55)	26.25	2.54 (2.42)	25.40	2.16 (2.17)	21.62	0.765
Plant Protein	1.28 (2.02)	12.84	0.78 (1.33)	7.75	1.29 (2.19)	12.90	1.95 (2.57)	19.46	0.132
SSB and Fruit Juice	5.16 (4.32)	51.63	4.93 (4.36)	49.25	5.99 (4.20)	59.90	5.43 (4.24)	54.32	0.279
Wholegrain	2.88 (2.94)	28.76	2.15 (2.81)	21.50	3.23 (2.92)	32.30	2.27 (2.71)	22.70	0.052
Red & Processed Meat	6.92 (2.99)	69.16	7.25 (3.19)	72.50	8.30 (2.63)	83.00	7.24 (3.17)	72.43	<0.001
Total n3polyunsaturated fat as total energy (%)	5.07 (2.05)	50.67	5.60 (2.32)	56.00	5.04 (2.02)	50.40	5.62 (2.30)	56.22	0.082
Polyunsaturated fat as total energy (%)	5.45 (2.52)	54.50	5.88 (1.92)	58.75	5.00 (1.42)	50.00	5.68 (2.40)	56.76	<0.001
Alcohol	6.20 (1.60)	62.04	6.80 (1.68)	68.00	6.90 (1.62)	69.00	6.86 (1.73)	68.65	0.158
Sodium	5.21 (2.38)	52.08	5.70 (2.58)	57.00	5.32 (2.50)	53.20	4.76 (2.37)	47.57	0.472
***Total Score: 0 (lowest)–100 (highest quality)***	***43.33 (11.23)***	***43.33***	***44.90 (12.86)***	***44.90***	***46.43 (9.87)***	***46.43***	***44.84 (11.08)***	***44.84***	***<0.001***
**United States (U.S.)**									
*AHEI-2010 Components*	*White*		*Black*		*Asian*		*Other*		
Score Range; 0 (lowest)–10 (highest quality)	Mean (SD)	Percent	Mean (SD)	Percent	Mean (SD)	Percent	Mean (SD)	Percent	P value
Fruit	2.03 (3.14)	20.344	1.85 (3.15)	18.51	3.61 (3.79)	36.09	2.87 (3.69)	28.70	<0.001
Vegetables	3.99 (3.50)	39.873	3.52 (3.47)	35.21	5.47 (3.62)	54.68	4.54 (3.60)	45.40	<0.001
Plant Protein	2.19 (3.40)	21.949	1.69 (3.18)	16.90	3.48 (4.10)	34.77	3.00 (3.88)	30.00	<0.001
SSB and Fruit Juice	7.09 (3.84)	70.879	7.27 (3.52)	72.75	9.03 (2.14)	90.32	7.24 (3.57)	72.40	<0.001
Wholegrain	2.34 (3.33)	23.42	1.84 (3.01)	18.42	3.45 (3.97)	34.49	1.88 (3.07)	18.80	<0.001
Red & Processed Meat	5.88 (3.90)	58.841	6.51 (3.82)	65.08	7.33 (3.57)	73.32	6.36 (3.91)	63.60	<0.001
EPA and DHA	1.86 (2.83)	18.637	2.49 (3.24)	24.86	3.00 (3.80)	30.04	2.19 (3.02)	21.90	<0.001
Polyunsaturated fat as total energy (%)	7.96 (1.71)	79.605	8.38 (1.58)	83.79	7.76 (1.66)	77.59	7.67 (1.69)	76.70	<0.001
Alcohol	4.94 (1.88)	49.363	4.93 (1.81)	49.25	4.93 (1.21)	49.34	4.84 (2.52)	48.40	0.975
Sodium	4.83 (2.29)	48.344	5.02 (2.46)	50.15	4.78 (2.34)	47.77	4.91 (1.77)	49.10	0.348
***Total Score: 0 (lowest)–100 (highest quality)***	***43.13 (12.22)***	***43.13***	***43.49 (11.23)***	***43.39***	***52.84 (11.34)***	***52.84***	***45.50 (12.09)***	***45.50***	***<0.001***

SSB, Sugar Sweetened Beverages; P value based on Kruskal-Wallis tests; EPA and DHA, Eicosapentaenoic acid and Docosahexaenoic acid.

**Table 2b.** DQI-I component scores across racial groups (U.K. and U.S.)

United Kingdom (U.K.)									
*DQI-I Components (Score Range; lowest-highest quality)*	*White*		*Black*		*Asian*		*Other*		P *value*
	Mean (SD)	Percent	Mean (SD)	Percent	Mean (SD)	Percent	Mean (SD)	Percent	
Food group variety (0–15)	7.51 (4.42)	50.06	5.85 (3.66)	39.00	7.89 (3.85)	52.60	7.05 (3.62)	47.03	0.049
Protein source variety (0–5)	1.28 (1.26)	25.51	0.95 (0.99)	19.00	1.34 (1.44)	26.80	1.43 (1.17)	47.75	0.336
Vegetables (0–5)	2.81 (1.94)	56.16	2.98 (2.04)	59.50	2.94 (1.87)	58.80	2.62 (2.02)	52.43	0.778
Fruit (0–5)	1.65 (1.98)	32.99	1.65 (2.14)	33.00	1.71 (1.93)	34.20	1.70 (1.98)	34.05	0.983
Wholegrains (0–5)	2.53 (2.23)	50.70	1.90 (2.23)	38.00	2.93 (2.20)	58.60	2.14 (2.03)	42.70	0.047
Fibre (0–5)	2.10 (1.61)	42.07	1.63 (1.60)	32.50	2.36 (1.49)	47.20	1.65 (1.69)	32.97	0.029
Protein (0–5)	4.71 (0.77)	94.11	4.65 (0.77)	93.00	4.53 (0.94)	90.60	4.95 (0.33)	98.92	0.022
Iron (0–5)	3.68 (1.56)	73.61	2.98 (2.04)	59.50	3.62 (1.54)	72.40	3.14 (1.78)	62.70	0.016
Calcium (0–5)	4.10 (1.28)	81.97	3.23 (1.53)	64.50	3.65 (1.40)	73.00	3.97 (1.36)	79.46	<0.001
Vitamin C (0–5)	4.40 (1.31)	87.97	4.63 (1.00)	92.50	4.52 (1.02)	90.40	4.41 (1.30)	88.11	0.784
Total Fat (0–6)	4.12 (2.29)	68.82	4.05 (2.21)	36.78	4.53 (2.02)	75.50	3.57 (2.63)	59.46	0.233
Saturated Fat (0–6)	3.43 (2.07)	57.21	4.05 (1.87)	31.11	4.47 (1.83)	74.50	3.97 (1.88)	66.22	<0.001
Cholesterol (0–6)	4.85 (2.04)	80.78	4.13 (2.51)	41.89	4.74 (2.22)	79.00	4.46 (2.41)	74.33	0.197
Sodium (0–6)	5.20 (1.58)	86.60	5.10 (1.69)	28.19	5.25 (1.67)	87.50	5.35 (1.25)	89.19	0.858
Empty Calorie Foods (0–6)	0.57 (1.47)	9.48	1.73 (2.34)	39.04	1.17 (1.70)	19.50	0.65 (1.44)	10.81	<0.001
Macronutrient ratio (0–6)	1.52 (1.36)	25.33	1.33 (1.57)	26.17	2.18 (1.85)	36.33	1.37 (1.28)	22.83	0.007
Fatty Acid ratio (0–6)	2.06 (0.98)	51.62	2.20 (1.16)	55.00	2.44 (1.03)	61.00	2.27 (0.90)	56.76	0.001
***Total Score: 0 (lowest)–100 (highest quality)***	***56.52 (13.10)***	***56.52***	***53.01 (11.56)***	***53.01***	***60.27 (11.92)***	***60.27***	***54.69 (11.82)***	***54.69***	***0.013***
**United States (U.S.)**									
*DQI-I Components (Score Range; lowest-highest quality)*	*White*		*Black*		*Asian*		*Other*		P *value*
	Mean (SD)	Percent	Mean (SD)	Percent	Mean (SD)	Percent	Mean (SD)	Percent	
Food group variety (0–15)	8.18 (3.39)	54.55	7.37 (3.34)	49.10	9.00 (3.09)	60.00	8.39 (3.39)	55.96	<0.001
Protein source variety (0–5)	1.66 (1.40)	33.15	1.50 (1.44)	29.96	1.72 (1.44)	34.30	1.95 (1.56)	38.96	<0.001
Vegetables (0–5)	0.75 (1.57)	14.92	0.72 (1.56)	14.47	1.48 (2.01)	29.58	1.12 (1.87)	22.36	<0.001
Fruit (0–5)	3.01 (2.12)	60.29	2.62 (2.18)	52.41	3.58 (1.98)	71.51	3.13 (2.10)	62.69	<0.001
Wholegrains (0–5)	0.78 (1.57)	15.67	0.60 (1.41)	12.06	1.41 (1.98)	28.11	0.62 (1.41)	12.34	<0.001
Fibre (0–5)	1.30 (1.64)	26.01	1.16 (1.61)	23.16	2.28 (1.89)	45.55	1.94 (1.78)	38.85	<0.001
Protein (0–5)	4.59 (0.94)	91.75	4.38 (1.18)	87.63	4.65 (0.85)	93.09	4.57 (0.95)	91.41	<0.001
Iron (0–5)	4.13 (1.50)	82.64	3.84 (1.69)	76.80	4.10 (1.49)	81.92	4.06 (1.52)	81.27	<0.001
Calcium (0–5)	3.09 (1.68)	61.75	2.41 (1.83)	48.13	2.55 (1.67)	50.94	2.96 (1.72)	59.26	<0.001
Vitamin C (0–5)	2.27 (2.14)	45.38	2.57 (2.15)	51.44	3.21 (1.99)	64.19	2.77 (2.15)	55.48	<0.001
Total Fat (0–6)	1.14 (1.52)	18.92	1.21 (1.57)	20.23	1.90 (1.72)	31.70	1.65 (1.70)	27.55	<0.001
Saturated Fat (0–6)	3.04 (1.96)	50.64	3.74 (1.91)	62.34	4.32 (1.83)	72.08	3.82 (1.98)	63.69	<0.001
Cholesterol (0–6)	4.11 (2.51)	68.54	3.98 (2.55)	66.26	4.11 (2.52)	68.49	3.96 (2.55)	66.00	0.296
Sodium (0–6)	2.45 (2.45)	40.86	2.73 (2.45)	45.56	2.43 (2.41)	40.47	2.69 (2.45)	44.87	0.006
Empty Calorie Foods (0–6)	0.79 (1.76)	13.19	0.87 (1.76)	14.53	2.00 (2.28)	33.30	1.26 (2.06)	20.96	<0.001
Macronutrient ratio (0–6)	1.61 (1.38)	26.88	1.67 (1.41)	27.89	1.90 (1.77)	31.75	1.75 (1.61)	29.18	0.309
Fatty Acid ratio (0–6)	2.28 (0.94)	57.04	2.57 (1.01)	64.37	2.55 (0.99)	63.63	2.42 (0.97)	60.59	<0.001
***Total Score: 0 (lowest)–100 (highest quality)***	***46.84 (11.56)***	***46.84***	***45.45 (11.51)***	***45.45***	***54.89 (12.55)***	***54.89***	***51.03 (12.44)***	***54.89***	***<0.001***

P value based on Kruskal-Wallis tests. Empty calorie foods include chocolate and non-chocolate confectionary, sugar sweetened beverages, and savoury snacks.

**Table 2c.** EAT-Lancet component scores across racial groups (U.K. and U.S.)

United Kingdom (U.K.)									
*EAT-Lancet Components*	*White*		*Black*		*Asian*		*Other*		
Score Range; 0 (lowest)–3 (highest)	Mean (SD)	Percent	Mean (SD)	Percent	Mean (SD)	Percent	Mean (SD)	Percent	P *value*
Vegetables	0.65 (0.82)	21.69	0.75 (0.93)	25.00	0.69 (0.90)	23.00	0.70 (0.85)	23.23	0.957
Fruit	1.10 (1.12)	36.62	1.13 (1.20)	37.50	1.19 (1.07)	39.67	1.18 (1.13)	39.39	0.732
Unsaturated oils	0.24 (0.54)	8.00	0.15 (0.43)	5.00	0.32 (0.62)	10.67	0.32 (0.66)	10.67	0.045
Legumes	0.82 (1.06)	27.45	0.55 (0.96)	18.33	0.67 (1.00)	22.33	0.85 (1.20)	28.28	0.170
Nuts	0.21 (0.62)	6.94	0.23 (0.70)	7.50	0.24 (0.67)	8.00	0.64 (1.11)	21.21	0.104
Wholegrains	0.15 (0.41)	5.15	0.05 (0.22)	1.67	0.14 (0.38)	4.67	0.06 (0.24)	2.02	0.428
Fish	1.16 (1.35)	38.50	1.38 (1.35)	45.83	1.08 (1.34)	36.00	1.79 (1.39)	59.60	0.058
Red & Processed Meat	1.12 (1.34)	37.46	1.08 (1.31)	35.83	1.79 (1.44)	59.67	1.52 (1.44)	50.51	<0.001
Pork	1.48 (1.32)	49.18	1.95 (1.26)	65.00	2.50 (1.04)	83.33	1.48 (1.42)	49.49	<0.001
Poultry	1.99 (1.07)	66.19	1.75 (1.13)	58.33	1.74 (1.18)	58.00	1.79 (0.99)	59.60	0.099
Eggs	1.83 (1.21)	60.86	1.75 (1.21)	58.33	1.69 (1.26)	56.33	1.42 (1.32)	47.47	0.327
Dairy	2.56 (0.76)	85.23	2.98 (0.16)	99.17	2.78 (0.60)	92.67	2.76 (0.56)	91.92	<0.001
Potatoes	2.49 (0.77)	82.89	2.88 (0.40)	95.83	2.82 (0.48)	94.00	2.72 (0.58)	90.63	<0.001
Added Sugar	1.89 (1.00)	63.00	2.05 (0.93)	68.33	2.21 (0.88)	73.67	2.03 (0.90)	67.67	0.457
***Total Score: 0 (lowest)–42 (highest)***	***17.67 (4.42)***	***42.07***	***18.65 (4.45)***	***44.05***	***19.86 (4.31)***	***47.29***	***18.92 (4.75)***	***45.05***	***<0.001***
**United States (U.S.)**									
*EAT-Lancet Components*	*White*		*Black*		*Asian*		*Other*		
Score Range; 0 (lowest)–3 (highest)	Mean (SD)	Percent	Mean (SD)	Percent	Mean (SD)	Percent	Mean (SD)	Percent	P value
Vegetables	1.40 (1.16)	46.67	1.22 (1.16)	40.67	1.71 (1.16)	57.11	1.43 (1.17)	47.82	<0.001
Unsaturated Oils	1.70 (1.02)	56.67	1.72 (1.07)	57.33	1.71 (0.99)	56.98	1.64 (1.03)	54.61	0.266
Legumes	0.51 (1.10)	17.00	0.42 (1.02)	14.00	1.00 (1.37)	33.33	0.99 (1.39)	33.05	<0.001
Nuts	0.67 (1.12)	37.33	0.47 (1.00)	15.67	0.84 (1.24)	27.86	0.50 (1.00)	16.57	<0.001
Wholegrains	0.19 (0.47)	6.33	0.14 (0.42)	4.67	0.37 (0.65)	12.26	0.14 (0.42)	4.82	<0.001
Fish	0.37 (0.97)	12.33	0.62 (1.19)	20.67	0.73 (1.26)	24.40	0.46 (1.06)	15.34	<0.001
Red & Processed Meat	0.15 (0.63)	5.00	0.28 (0.84)	28.00	0.50 (1.09)	16.73	0.24 (0.79)	8.07	<0.001
Poultry	2.31 (1.07)	77.00	1.97 (1.23)	65.67	2.12 (1.16)	70.63	2.13 (1.18)	70.86	<0.001
Eggs	2.25 (1.15)	75.00	2.34 (1.09)	78.00	2.20 (1.15)	73.33	2.15 (1.22)	71.74	0.003
Pork	0.40 (1.00)	13.33	0.55 (1.14)	18.33	0.64 (1.22)	21.45	0.42 (1.02)	14.06	<0.001
Dairy	1.17 (1.22)	39.00	1.65 (1.28)	55.00	1.89 (1.20)	63.02	1.37 (1.25)	45.79	<0.001
Potatoes	2.30 (1.03)	76.67	2.35 (0.99)	78.33	2.55 (0.81)	85.16	2.49 (0.88)	83.01	<0.001
Added Sugar	1.66 (1.07)	55.33	1.54 (1.07)	51.33	2.18 (0.91)	72.64	1.73 (1.07)	57.79	<0.001
Fruit	0.52 (0.88)	17.33	0.48 (0.89)	16.00	0.96 (1.08)	32.01	0.77 (1.04)	25.58	<0.001
***Total Score: 0 (lowest)–42 (highest)***	***15.61 (4.13)***	***37.17***	***15.76 (4.53)***	***37.52***	***19.41 (5.11)***	***46.21***	***16.47 (4.35)***	***39.21***	***<0.001***

P value based on Kruskal-Wallis tests.

### Diet quality across racial groups (within countries)

**Fig. 1.**
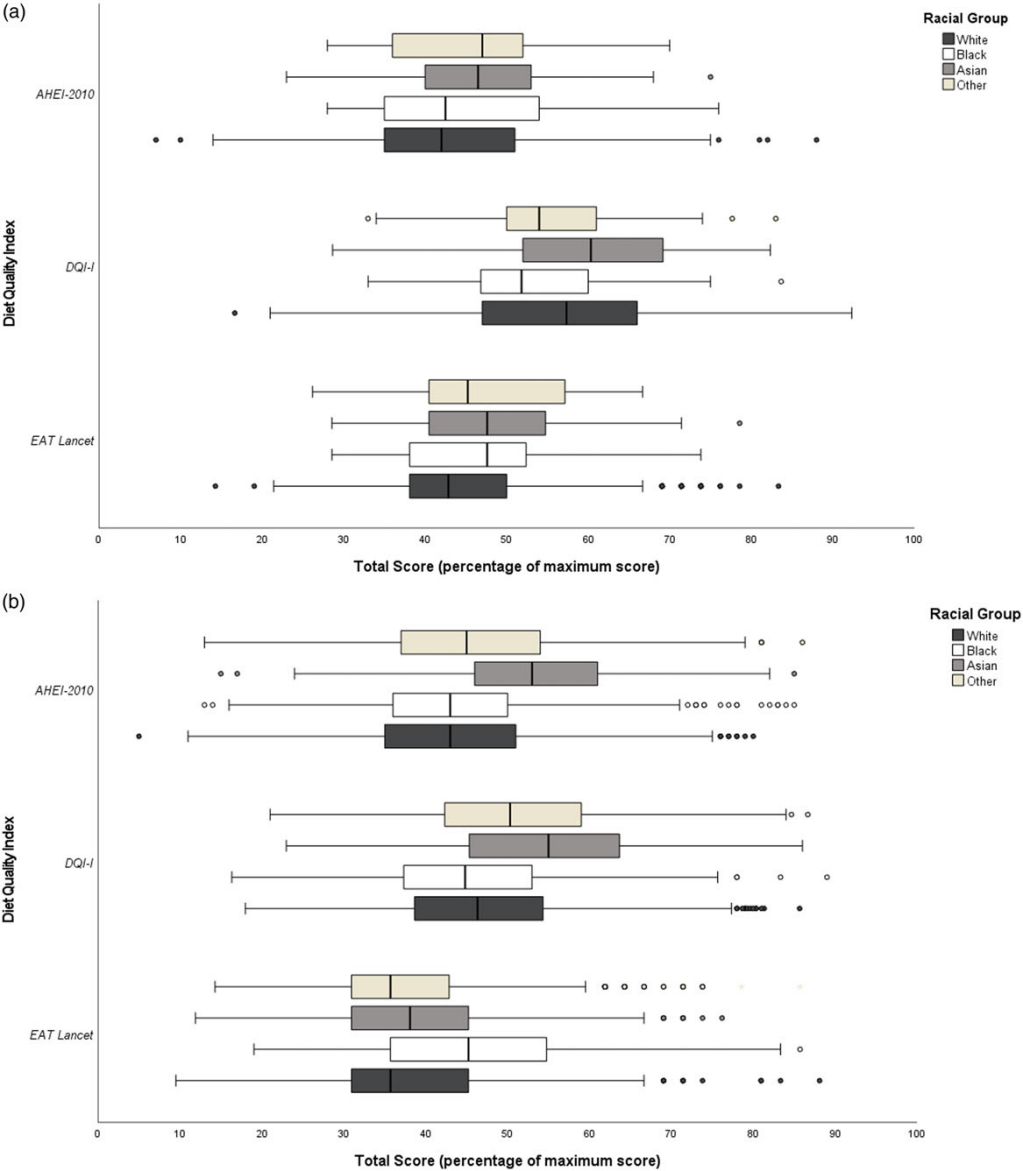
Median diet quality scores (as percentage of maximum score) and ANCOVA across U.K. racial groups. AHEI-2010 = Alternative Healthy Eating Index (2010), DQI-I = Diet Quality Index-International, * = P < 0.05, ** = P < 0.001. (a) adjusted for age, sex, income, and education with Bonferroni pairwise comparisons corrected P value shown. (b) Only adjusted for age, sex, income, and education with Bonferroni pairwise comparisons corrected P value shown.

Median total scores of AHEI-2010, DQI-I, and EAT Lancet indices of U.K. and U.S. racial groups are illustrated in Fig. 1a and b respectively, presented as a percentage of the maximum total score of each index due to differing index scoring. In all indices a higher percentage indicates better adherence to recommendations and higher diet quality.

Among U.K. racial groups, median AHEI-2010 scores (as a percentage of maximum total) ranged from 42 to 47%, where White AHEI-2010 scores were significantly lower than Asian AHEI-2010 (median scores of 42 and 47 respectively), P < 0.001 once adjusted for demographics and Bonferroni pairwise comparisons (Fig. 1a). Significantly lower EAT-Lancet median scores were found for the U.K. White group compared to the U.K. Asian group (43% *vs*. 48% of maximum total score). The U.K. White group had higher DQI-I median scores than Black and Other cohorts, with significant differences observed across White and Black groups in the fully adjusted model (P < 0.001). Asian groups achieved the highest median total scores for the AHEI-2010, DQI-I, and EAT-Lancet indices, however scores were not significantly higher than Black or Other cohorts in fully adjusted models. In the U.S., median total index scores were highest amongst the Asian cohort who achieved 53%, 55% and 45% of maximum total scores for AHEI-2010, DQI-I and EAT Lancet index respectively (Fig. 1b). Larger differences across racial groups were observed in the U.S. than the U.K. U.S. Asian and Other groups achieved significantly higher AHEI-2010, DQI-I, and EAT-Lancet index total scores (P < 0.001 once adjusted for demographics and Bonferroni pairwise comparisons) than White and Black groups, while the Other group achieved significantly lower median total AHEI-2010, DQI-I, and EAT-Lancet index scores than the Asian group in the U.S.

### Diet quality across racial groups (across countries)

**Fig. 2.**
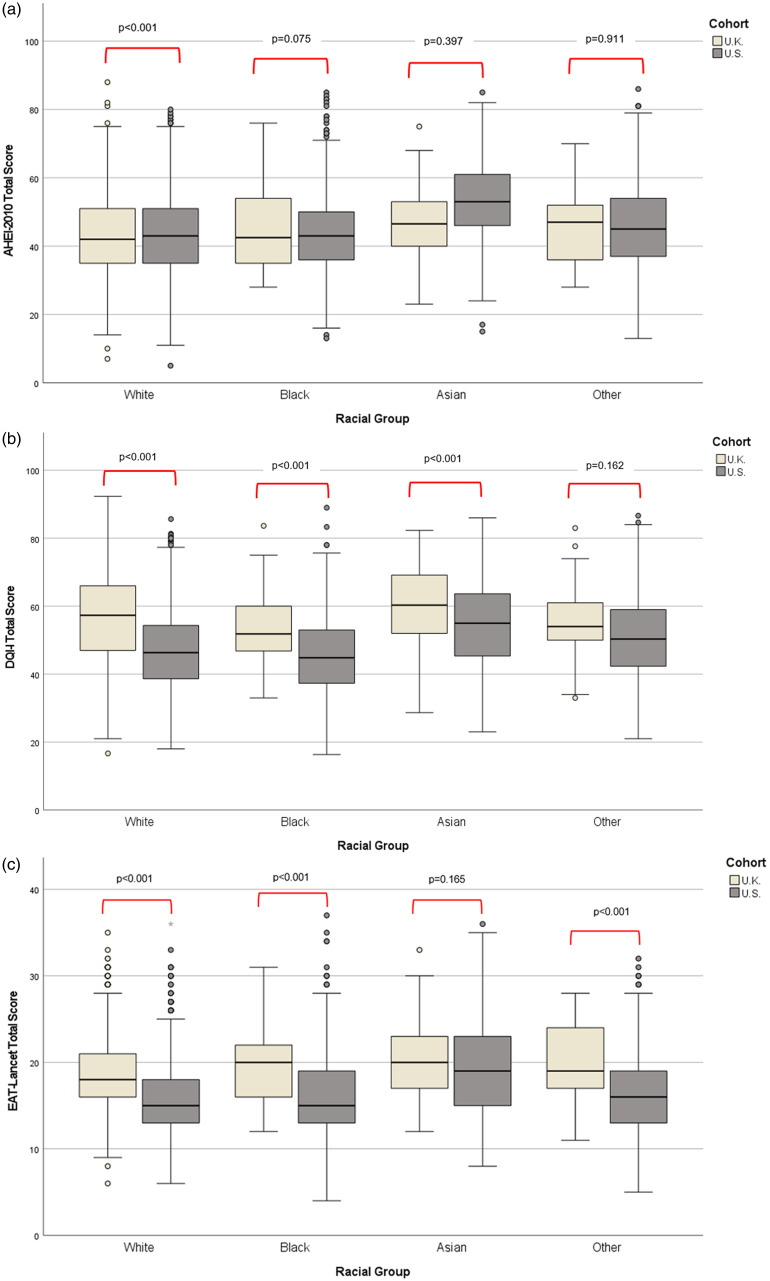
(a) Median AHEI-2010 total scores and ANCOVA across racial groups in the U.K. vs. U.S. AHEI-2010 = Alternative Healthy Eating Index (2010), adjusted for age, sex, income, and education with Bonferroni pairwise comparisons corrected P value shown. (b) Median DQI-I total scores and ANCOVA across racial groups in the U.K. vs. U.S. DQI-I = Diet Quality Index International, adjusted for age, sex, income, and education with Bonferroni pairwise comparisons corrected P value shown. (c) Median EAT-Lancet total scores and ANCOVA across racial groups in the U.K. vs. U.S. Adjusted for age, sex, income, and education with Bonferroni pairwise comparisons corrected P value shown.

Median total diet quality scores obtained by racial groups in the U.K. were compared to the those of the same racial group in the U.S. (Fig. 2a–c).

Similar trends were noted across countries, where Asian participants from the U.K. and U.S. had highest median total index scores across all indices examined. When racial groups were considered across location individually, diet quality of U.S. racial groups, as estimated by DQI-I and EAT-Lancet, was lower than the same racial group in the U.K. The U.K. White group reported significantly higher median total scores for dietary adequacy (57 vs. 47; Fig. 2b) and sustainability (19 vs. 16; Fig. 2c) than their U.S. counterparts. U.K. Black and Other groups also achieved significantly higher median EAT-Lancet scores than their U.S. counterparts (Fig. 2c). U.K. Black median DQI-I total scores were also significantly higher than U.S. Black DQI-I scores (51 vs. 45; P < 0.001). Significant differences across U.K. and U.S. Asian median diet quality scores were noted for DQI-I (60 vs. 55; P < 0.001). Diet quality as calculated by AHEI-2010 was higher in U.S. White, Black and Asian racial groups than their U.K. counterparts (Fig. 2a).

### Index component scores

Many dietary components of each index (AHEI-2010, DQI-I, and EAT-Lancet; Tables 2a–2c respectively) followed the same trend across racial groups, with highest individual component scores often achieved by Asian groups, while lowest scores were most often reported within White and Black groups. Larger significant differences were reported across U.S. racial groups than U.K. groups. Despite significant differences in component scores across racial groups, low component scores of similar food groups were consistently observed across racial group and diet quality index, namely wholegrain, fruit, vegetable (U.K. only), and a variety of protein sources. Components which largely differed across racial groups are outlined in greater detail for each diet quality index below.

#### AHEI-2010

In the U.K. only the components ‘Red and Processed Meat’ and ‘Polyunsaturated fat as total energy’ differed significantly across groups and scores were lowest in White (6.92/10) and Asian (5.00/10) respectively (Table 2a). Among the U.S. cohort, significant differences were observed across all AHEI-2010 components except ‘Alcohol’ and ‘Sodium’. Asian mean scores were highest for all remaining components except for ‘Polyunsaturated fat as total energy’ which was highest amongst the Black group, as found within the U.K. dataset (U.S. Black mean score = 8.39; P < 0.001). Mean scores obtained by U.S. Asians for ‘Wholegrain’ and ‘Sugar Sweetened Beverages and Fruit Juice’ (SSB) were most notably higher than remaining racial groups (wholegrain mean score: Asian = 3.45 *vs*. White = 2.34, Black = 1.84 and Other = 1.88, P < 0.001; SSB mean score: Asian = 9.03 *vs*. White = 7.09, Black = 7.27 and Other = 7.24, P < 0.001) (Table 2a).

#### DQI-I

In total, 10 of the 17 DQI-I components differed significantly across U.K. racial groups and in 8 of these 10 components, lowest scores were reported by the Black group (Table 2b). Despite high protein scores across all racial groups (>90% obtained maximum protein score) protein source variety was lowest among Black, followed by White and Asian groups (mean scores of 0.95, 1.28 and 1.34, scored out of 5, respectively). U.K. Black groups obtained significantly lower nutrient scores than remaining U.K. racial groups for ‘sodium’, ‘calcium’, and fat (‘total fat’, ‘saturated fat’ and ‘cholesterol’) achieving ≥9% less of the total maximum score for each component than their U.K. counterparts. In the U.S., the Black group achieved the lowest mean score for 10 out of the 16 DQI-I components where significant differences were reported across racial groups. Similar to the U.K., the Black group in the U.S. achieved lowest protein source variety (1.50/5) and calcium (2.41/5) and iron (3.84/5) scores (P < 0.001) (Table 2b). However, the U.S. Black group obtained slightly higher scores (i.e. lower intake) for ‘total fat’, ‘saturated fat’ and ‘sodium’ than their White counterparts, but scores remained significantly lower than Asian and Other groups.

#### EAT-lancet

Component scores for ‘Unsaturated Oils’, ‘Red and Processed Meat’, ‘Pork’, ‘Dairy’ and ‘Potatoes’ differed significantly across U.K. racial groups. Highest scores obtained for these EAT-Lancet components varied across races. Mean score of unsaturated oils was highest among Asian and Other groups and lowest among Black groups (Asian and Other: 0.32 *vs*. Black: 0.15; P = 0.005); highest mean ‘Red and Processed Meat’ and ‘Pork’ scores was obtained by the Asian group (1.79; P < 0.001 and 2.50; P < 0.001 respectively); Black groups achieved the highest mean score for ‘Dairy’ and ‘Potatoes’ but scores were similar to Asian and Other groups, the White group had the lowest mean score indicating high intake of dairy and potatoes (Table 2c). Significantly different mean scores were observed for all components except ‘Unsaturated Oils’ across U.S. racial groups. The Asian group obtained the highest scores for all EAT-Lancet components except ‘Poultry’ where the White group reported the highest score (i.e. lowest intake) (2.31 *vs*. 1.97, 2.12 and 2.13 by White, Black, Asian, and Other groups respectively; P < 0.001) (Table 2c). The U.S. Asian group had significantly higher scores for ‘Wholegrain’, ‘Red and Processed Meat’ and ‘Added Sugar’ than all other racial groups and significantly higher scores ‘Fish’ and ‘Dairy’ than the White group.

### Association across indices

Spearman correlations reported moderate associations between AHEI-2010 and DQI-I scores among racial groups in both the U.K. and U.S.; medium-high correlations were found for White and Asian groups in the U.K. and the U.S. (*r* = 0.45–0.65; P < 0.001; Table 3), however moderate-low correlations were reported for U.K. Other and U.S. Black groups. Moderate-high correlations were reported between AHEI-2010 and EAT-Lancet scores across, and correlations were similar across all racial groups in both countries (*r* = 0.53–0.65; P < 0.001; Table 3). Associations between DQI-I and EAT-Lancet differed across racial groups and by country. Again correlations across indices were similar within White and Asian groups in both countries (moderate-high); correlations within Black and Other differed across countries (Table 3).

**Table 3.** Spearman correlations across index scores among U.K. and U.S. racial groups

	AHEI-2010 (/100)	DQI-I (/100)	EAT-Lancet (/42)	AHEI-2010 *vs.* DQI-I	AHEI-2010 *vs.* EAT Lancet	DQI-I *vs*. EAT Lancet
Racial Group	Median (IQR)	Median (IQR)	Median (IQR)	*r,* P value	*r,* P value	*r,* P value
*United Kingdom (U.K.)*						
White	42.00 (16.00)	57.33 (19.00)	18.00 (5.00)	0.50^a^	0.58^a^	0.35^a^
Black	42.50 (19.00)	51.83 (13.59)	20.00 (6.00)	0.51^a^	0.57^a^	0.40 (0.01)
Asian	46.50 (13.00)	60.33 (17.41)	20.00 (6.00)	0.46^a^	0.53^a^	0.43^a^
Other	47.00 (16.50)	54.00 (12.84)	19.00 (7.00)	0.28	0.65^a^	0.21
*United States (U.S.)*						
White	43.00 (16.00)	46.33 (15.67)	15.00 (5.00)	0.45^a^	0.58^a^	0.36^a^
Black	43.00 (14.00)	44.83 (15.67)	15.00 (6.00)	0.39^a^	0.56^a^	0.27^a^
Asian	53.00 (15.25)	55.00 (18.41)	19.00 (8.00)	0.56^a^	0.65^a^	0.53^a^
Other	45.00 (17.00)	50.32 (16.67)	16.00 (6.00)	0.56^a^	0.59^a^	0.46^a^

IQR, interquartile range.

a =P < 0.001.

## Discussion

Analysing quality of habitual intake is a useful strategy to monitor population adherence to public health advice.^(45–47)^ However insight into the quality of dietary intake across different cultural groups is limited despite the increased prevalence of diverse racial and ethnic groups living in developed regions worldwide.^(14)^ This secondary analysis of recent NDNS and NHANES data demonstrates that while differences exist across racial groups, these differences follow similar trends by country and diet quality index. Highest quality in terms of healthiness, dietary adequacy and sustainability were reported by Asian groups in the U.K. and U.S., with more sustainable intake – as defined by EAT-Lancet - reported among U.K. racial groups. Greater adherence to recommendations for wholegrains, vegetables and legumes, red and processed meat intake would improve quality of U.K. and U.S. population diets as a whole.

To contextualise these results, this research was compared to similar work examining diet quality in U.K. and U.S. populations. A worldwide analysis of diet quality described a global mean AHEI score as *‘modest’,* where Asian populations reported higher diet quality (AHEI) compared to U.S. and South American populations.^(48)^ Similarly, a higher DQI-I score was reported among Chinese adults compared to U.S adults., with better vegetable, wholegrain and fatty acid contributing to higher diet quality among the Chinese group.^(11)^ Previous insights into diet quality of U.S. adults using NHANES data reported a mean Healthy Eating Index (HEI) score of 58, with scores differing across age groups, lowest among ages 14–18 years.^(15)^ A recent report by Tao *et al.,* examined diet quality trends specifically within racial groups in the U.S. In agreement with results presented here, the analysis reported highest total HEI scores among Asian adults, whose quality of intake has remained stable between 2011 and 2018.^(16)^ Diet quality was poorest among Black adults and worryingly, HEI scores of White and Black groups are on a downward trajectory since 2011. Examining intake of individual HEI components, poor adherence to legumes, fruit and wholegrains contributed to suboptimal total scores.^(16)^ Similarly in the U.K., low intake of wholegrains and legumes and high intake of processed meat has been noted, contributing to moderate total diet quality scores (HEI-2015 score of 60, AHEI-2010 score of 50) among U.K. adults.^(49)^

As demonstrated by this investigation, similar improvements to dietary patterns, for example reducing red/processed meat and increasing vegetable consumption, will not only enhance adherence to dietary and healthy eating guidelines but also improve the sustainability of food intake. This research has identified that there is no specific dietary component which is of significant concern in one racial group and not another. Across the board an increase in wholegrain, fruit, vegetable, fish, and legume intake and a reduction of red and processed meat would improve adherence to current food-based dietary guidelines, which have long been cited as key dietary components whose intake does not align to recommendations, across many population groups.^(18,46)^ However, it is evident from this and previous research that nutrition adequacy does vary across racial groups and group-specific intakes must be considered when developing and implementing dietary advice.^(14)^ Stronger adherence to existing food-based dietary guidelines would reduce green-gas emissions caused by diet by approximately 25% without the need for total elimination of food groups, meaning inadequacies of nutrients primarily sourced from animal produce (e.g. vitamin B12 and calcium) would not be of serious public health concern moving forward.^(50,51)^ Furthermore, promoting food behaviour change such as limiting food waste would further reduce the carbon footprint of agri-food systems without risking the dietary adequacy and health of vulnerable minority groups within a country.^(52,53)^

It is true that similar changes in dietary patterns are needed by U.K. and U.S. racial groups, nonetheless, some groups may need more targeted support than others to adhere to recommendations; generic messaging like ‘increase consumption of plant-based food’ will not provide a platform to address dietary inequalities.^(14)^ High diet quality has been consistently reported among Asian groups relative to other racial groups, both here and elsewhere.^(16,18,48,54)^ More research is needed to understand the key drivers of specific dietary patterns and the extent to which food choice influences vary across racial groups in order to mitigate the potential racial health disparities resulting from poor dietary intake.^(14,18,55)^ Understanding taste and cooking preferences and incorporating more ethnic foods into sample healthy and sustainable recipes/food pyramids could undoubtedly help encourage dietary change among minority racial groups.^(56–58)^ As highlighted by this research, the physical environment may be integral to diet quality of populations, where cultural and social factors have long been associated with dietary intake and quality of intake.^(18,59–61)^ Deeper knowledge of how social and environmental factors impact dietary patterns across racial groups would provide greater detail of how public guidance and initiatives could encourage healthy and sustainable intake across population subgroups most in need.^(62)^ Until social and environmental influences are fully understood across all population subgroups, improving dietary adequacy, healthiness and sustainability of diets of minority groups would be extremely challenging.

## Strengths and limitations

To the researchers’ knowledge, this is the first paper to investigate diet quality of racial groups, examining the dietary adequacy, disease risk and sustainability of dietary intake in combination across the U.K. and U.S. Previously validated dietary indices were applied to national dietary intake data: AHEI-2010 – previously associated with favourable health outcomes including cardiometabolic parameters and lower rates of chronic disease incidence and mortality^(12,29,63)^; DQI-I – shown to facilitate cross-country comparisons of diet quality, allowing flexibility to apply country-specific recommendations^(11,64)^ and EAT-Lancet (Stubbendorf *et al.,* 2022 approach) – determining adherence to EAT-Lancet recommendations and associated with mortality risk.^(13,36)^

Some limitations of this work must be noted. Not all components assessed in AHEI-2010, DQI-I, and EAT-Lancet indices were available in NDNS and NHANES datasets, resulting in estimation of certain dietary component scores and alternations to AHEI-2010 were necessary. In addition, very poor distribution of participants across racial groups was achieved in the NDNS dataset (however were representative of U.K. census data) and although findings complement those reported across U.S. racial groups interpretation of results must be cautioned. In future national dietary surveys oversampling of minority groups (as done for NHANES 2017–2018 data) may be advisable to ensure accurate and meaningful analysis can be performed on data collected from population subgroups.

## Conclusion

Variances in diet quality were observed across racial groups in the U.K. and U.S., White groups exhibited poorest adherence to EAT-Lancet recommendations and Asian groups demonstrated strongest adherence to healthy and sustainable diets. Unfavourable intake of wholegrain, fruit, vegetables/legumes, and processed meats was consistently reported by racial groups however better adherence to intake recommendations were found among Asian and Other groups than Black and White groups in both countries. This research highlights that to promote optimum healthy and sustainable dietary intake across all subgroups of the population equally, current dietary patterns of population subgroups need to be fully understood so that diversity in consumption across cultural groups can be adequately addressed where required. In the future, food-based dietary guidelines should not only be developed with dietary adequacy and planetary health in mind, but also consider the individual needs of population subgroups, tailoring recommendations to specific cultures where appropriate.

## Supporting information

Bennett and Gibney supplementary material Bennett and Gibney supplementary material

## Supplementary material

The supplementary material for this article can be found at https://doi.org/10.1017/jns.2024.64.
