# Supplementary material for: An investigation of diet quality across racial groups in the United Kingdom and United States considering nutritional adequacy, disease risk, and environmental sustainability: a secondary analysis of NDNS and NHANES datasets
Source: J Nutr Sci. 2024 Dec 13;13:e93. doi: 10.1017/jns.2024.64 (PMC11658946; doi:10.1017/jns.2024.64)
Supplement: Bennett and Gibney supplementary material [file S2048679024000648sup001.docx]

***Supplementary Table 1a:*** *Gradual scoring approach applied to AHEI-2010*

| **Alternative Healthy Eating Index (AHEI-2010) Component Scores** | | | | | | | | | | | |
| --- | --- | --- | --- | --- | --- | --- | --- | --- | --- | --- | --- |
| *AHEI-2010 Score* | *0* | *1* | *2* | *3* | *4* | *5* | *6* | *7* | *8* | *9* | *10* |
| Fruit (servings/day) | 0 | 0.4 | 0.8 | 1.2 | 1.6 | 2 | 2.4 | 2.8 | 3.2 | 3.6 | 4+ |
| Vegetables (servings/day) | 0 | 0.5 | 1 | 1.5 | 2 | 2.5 | 3 | 3.5 | 4 | 4.5 | 5+ |
| Plant Protein (oz/day) | 0 | 0.1 | 0.2 | 0.3 | 0.4 | 0.5 | 0.6 | 0.7 | 0.8 | 0.9 | 1+ |
| SSB and FJ (oz/day) | 1+ | 0.9 | 0.8 | 0.7 | 0.6 | 0.5 | 0.4 | 0.3 | 0.2 | 0.1 | 0 |
| Wholegrains F (g/day) | 0-7.50 | 7.51-15.00 | 15.01-22.50 | 22.51-30.00 | 30.01-37.50 | 37.51-45.00 | 45.01-52.50 | 52.51-60.00 | 60.01-67.50 | 67.51-74.99 | ≥75 |
| Wholegrains M (g/day) | 0-8.99 | 9-17.99 | 18-26.99 | 27-35.99 | 36-44.99 | 45-53.99 | 54-62.99 | 63-71.99 | 72-80.99 | 81-89.99 | ≥90 |
| Red Meat (servings/day) | >1.50 |  | 1.21-1.50 |  | 0.91-1.20 |  | 0.61-0.90 |  | 0.31-0.60 |  | 0-0.30 |
| DHA+EPA (mg/day)* | 0-24.99 | 25-49.99 | 50-74.99 | 75-99.99 | 100-124.99 | 125-149.99 | 150-174.99 | 175-199.99 | 200-224.99 | 225-249.99 | ≥250 |
| N3polyunsaturated fat as total energy** (%) | 0-0.50 |  |  |  | 0.51-1.00 |  | 1.01-1.50 |  | 1.51-2.00 |  | >2.00 |

| **Alternative Healthy Eating Index (AHEI-2010) Component Scores** | | | | | | | | | | | |
| --- | --- | --- | --- | --- | --- | --- | --- | --- | --- | --- | --- |
| *AHEI-2010 Score* | *0* | *1* | *2* | *3* | *4* | *5* | *6* | *7* | *8* | *9* | *10* |
| Alcohol F (servings/day) | >2.50 |  |  |  |  | All other intake |  |  |  |  | 0.50-1.50 |
| Alcohol M (servings/day) | >3.50 |  |  |  |  | All other intake |  |  |  |  | 1.50-2.50 |
| Polyunsaturated fat as total energy (%) | 0-2 |  |  | >2-4.5 |  |  | >4.5-6.5 |  | >6.5-9.99 |  | ≥10 |

oz/day=ounces per day, SSB=sugar sweetened beverage, FJ=fruit juice, F=female, M=male *=applied to NHANES data, EPA & DHA=Eicosapentaenoic acid and Docosahexaenoic acid, **=applied to NDNS data, non-alcohol consumers were given a standardised score of 5, sodium scores based on decile of sodium intakes (mg/day) and were based on 1. U.K. females, 2. U.K. males, 3. U.S. females, 4. U.S. males.

***Supplementary Table 1b:*** *Gradual scoring applied to DQI-I*

| **Diet Quality International-Index (DQI-I) Component Scores** | | | | | | |
| --- | --- | --- | --- | --- | --- | --- |
| *DQI-I Scores* | *0* | *3* | *6* | *9* | *12* | *15* |
| Food group variety (number/day) | ≤100.00 | 100.01-200.00 | 200.01-300.00 | ≥300.01 |  |  |
|  | *0* | *1* | *3* | *5* | | |
| Food group variety protein (number/day) | ≤50.00 | 50.01-100.00 | 100.01-200.00 | ≥200.01 | | |
|  | *0* | *3* | *5* | | | |
| Vegetable (servings/day) | 0-49% RDA | 50-99% RDA | ≥100% RDA | | | |
| Fruit (servings/day) | 0-49% RDA | 50-99% RDA | ≥100% RDA | | | |
| Grain (servings/day) | 0-49% RDA | 50-99% RDA | ≥100% RDA | | | |
| Fibre (g/day) | 0-49% RDA | 50-99% RDA | ≥100% RDA | | | |
| Protein (g/day) | 0-49% RDA | 50-99% RDA | ≥100% RDA | | | |
| Iron (mg/day) | 0-49% RDA | 50-99% RDA | ≥100% RDA | | | |
| Calcium (mg/day) | 0-49% RDA | 50-99% RDA | ≥100% RDA | | | |
| Vitamin C (Mg/day) | 0-49% RDA | 50-99% RDA | ≥100% RDA | | | |
|  | *0* | *3* | *6* | | | |
| Total Fat (% energy) | >40 | >35 | <35 | | | |
| Saturated Fat (% energy) | >16 | >11 | <11 | | | |

| **Diet Quality International-Index (DQI-I) Component Scores** | | | |
| --- | --- | --- | --- |
| *DQI-I Scores* | *0* | *3* | *6* |
| Cholesterol (mg/day) | >400 | >300-400 | ≤300 |
| Sodium (mg/day) | >3400 | >2400-3400 | ≤2400 |
| Empty calorie foods (% energy) | >10 | >3-10 | ≤3 |
|  | *0* | *4* | *6* |
| Macronutrient ratio | Other | If CHO (52-68.9), Protein (9-16), Fat (13-27) | If CHO (55-65), Protein (10-15), Fat (15-25)  If CHO (50-70), Protein (8-17), Fat (12-30) |
|  | *0* | *2* | *4* |
| Fatty acid ratio | Other | If PUFA/SFA=0.8-1.7  If MUFA/SFA=0.8-1.7 | If PUFA/SFA=1-1.5  IF MUFA/SFA=1-1.5 |

%=percent, RDA=recommended daily allowance, CHO=cholesterol, PUFA=polyunsaturated fatty acids, SFA=saturated fat, MUFA=monounsaturated fatty acids.

***Supplementary Table 1c:*** *Gradual scoring approach applied to EAT-Lancet*

| **EAT-Lancet Component Scores** | | | | |
| --- | --- | --- | --- | --- |
| *EAT-Lancet Score* | *0* | *1* | *2* | *3* |
| Vegetables (g/day) | ≤100.00 | 100.01-200.00 | 200.01-300.00 | ≥300.01 |
| Fruit (g/day) | ≤50.00 | 50.01-100.00 | 100.01-200.00 | ≥200.01 |
| Unsaturated Oils (g/day) | ≤10.00 | 10.01-20.00 | 20.01-40.00 | ≥40.01 |
| Legumes (g/day) | ≤18.75 | 18.76-37.50 | 37.51-75.00 | ≥75.01 |
| Nuts(g/day) | ≤12.50 | 12.51-25.00 | 25.01-50.00 | ≥50.01 |
| Wholegrains (g/day) | ≤58.00 | 58.01-116.00 | 116.01-232.00 | ≥232.01 |
| Fish (g/day) | ≤7.00 | 7.01-14.00 | 14.01-28.00 | ≥28.01 |
| Red Meat (g/day) | ≥28.01 | 14.01-28.00 | 7.00-14.00 | ≤6.99 |
| Pork (g/day) | ≥28.01 | 14.01-28.00 | 7.00-14.00 | ≤6.99 |
| Poultry (g/day) | ≥116.01 | 58.01-116 | 29.00-58.00 | ≤28.99 |
| Eggs (g/day) | ≥50.01 | 25.01-50.00 | 13.00-25.00 | ≤12.99 |
| Dairy (g/day) | ≥1000.01 | 500.01-1000.00 | 250.00-500.00 | ≤249.99 |
| Potatoes (g/day) | ≥200.01 | 100.01-200.00 | 50.00-200.00 | ≤49.99 |
| Added Sugar (g/day) | ≥124.01 | 62.01-124.00 | 31.00-62.00 | ≤30.99 |

***Supplementary Table 2:*** *Adaptations to NDNS/NHANES variables made to allow estimation of diet quality component scores*

| **Diet Quality Index** | **Component** | **NDNS** | **NHANES** |
| --- | --- | --- | --- |
| AHEI-2010 | Trans-fat | Not available in NHANES dataset, not considered in AHEI-2010 scoring of NDNS cohort to allow direct comparison with NHANES cohort. | Trans-fat was not available in NHANES dataset, and no suitable substitute was available. Therefore, trans-fat component was removed from AHEI-2010 scoring of NHANES cohort [11]. |
|  | DHA+EPA | In the NDNS dataset, total n3 polyunsaturated fatty acid intake was reported and was not separated further. As a result, intake of DHA and EPA only could not be calculated. Instead, to estimate polyunsaturated intake, total n3-polyunsaturated fatty acid as a percentage of total energy was calculated and scored based on adherence to the World Health Organization (WHO) and FAO recommendations [26]. If total n3-polyunsaturated fatty acid intake ≥10% total energy, the maximum component score of 10 was given. | DHA and EPA available as separate variables in NHANES dataset. Individual DHA and EPA reported intakes were combined and a new DHA+EPA variable was created. Total n3 polyunsaturated fatty acid intake was not available in the NHANES dataset and therefore the same approach applied to NDNS data could not have been applied to NHANES data. |
|  | Wholegrain | Wholegrain intake was estimated based on intake of relevant NDNS food categories; “brown, granary and wheatgerm bread”, “wholemeal bread” and “high fibre cereals”. Total grams consumed of each of these food categories were summed and percentage wholegrain content per gram consumed, based on Jones *et al.,* 2017, were applied to estimate total wholegrain intake [27, 28]. | Wholegrain (grams/day) intake was available for NHANES cohort and no changes were necessary. |

| **Diet Quality Index** | **Component** | **NDNS** | **NHANES** |
| --- | --- | --- | --- |
| *AHEI-2010* | Alcohol | To determine the total number of alcohol servings consumed, U.K. standard units for beer, wine, and spirits were applied to grams of each alcohol type consumed and then total servings from each drink category were summed together to compute total alcohol servings [29]. | Alcohol (units/day) intake was available for NHANES cohort and no changes were necessary. |
| *DQI-I* | Empty Calorie Foods | To estimate intake of NDNS cohort, total consumption of chocolate and non-chocolate confectionary, savoury snacks, desserts, sugar-sweetened beverages, and alcohol were combined. Contribution of “empty calorie foods” intake to total energy intake was then calculated to determine “empty calorie foods” component score; ≤3% of total energy=6, >3-10%=3 and 10%=0. | To estimate intake of NHANES cohort, total consumption of chocolate and non-chocolate confectionary, savoury snacks, desserts, sugar-sweetened beverages, and alcohol were combined. Contribution of “empty calorie foods” intake to total energy intake was then calculated to determine “empty calorie foods” component score; ≤3% of total energy=6, >3-10%=3 and 10%=0. |
|  | Wholegrain | Wholegrain intake was estimated based on intake of relevant NDNS food categories; “brown, granary and wheatgerm bread”, “wholemeal bread” and “high fibre cereals”. Total grams consumed of each of these food categories were summed and percentage wholegrain content per gram consumed, based on Jones *et al.,* 2017, were applied to estimate total wholegrain intake [27, 28]. | Wholegrain (grams/day) intake was available for NHANES cohort and no changes were necessary. |

| **Diet Quality Index** | **Component** | **NDNS** | **NHANES** |
| --- | --- | --- | --- |
| *EAT-Lancet* | Beef and Lamb | All red meat and processed red meat were included in this component. | All red meat and processed red meat were included in this component. |
|  | Wholegrain | Wholegrain intake was estimated based on intake of relevant NDNS food categories; “brown, granary and wheatgerm bread”, “wholemeal bread” and “high fibre cereals”. Total grams consumed of each of these food categories were summed and percentage wholegrain content per gram consumed, based on Jones *et al.,* 2017, were applied to estimate total wholegrain intake [27, 28]. | Wholegrain (grams/day) intake was available for NHANES cohort and no changes were necessary. |
|  | Added Sugar | To calculate “added sugar” component scores of U.K. participants, relevant NDNS food categories containing added sugar were identified (e.g., “soft drinks”) and percentage contribution of added sugar to total weight of these food categories were estimated based on McCance and Widdowson retail data [32-34]. These percentages were then applied to total intake of relevant NDNS food categories to estimate the amount of added sugar consumed in grams. |  |
|  | Unsaturated Oils | Within NDNS data, the “unsaturated oils” component was calculated based on the combined intake (in grams per day) of corresponding NDNS food categories; “polyunsaturated margarine and oils”, “low fat spreads” and “reduced fat spreads (polyunsaturated only)”. |  |

***Supplementary Table 3a:*** *Demographic characteristics by tertiles of AHEI-2010 scores (U.K. vs. U.S.)*

|  | | **AHEI-2010 (U.K.)** | | | | **AHEI-2010 (U.S.)** | | | |
| --- | --- | --- | --- | --- | --- | --- | --- | --- | --- |
| Tertile (Score range) | | 1 (0-37.99) | 2 (38-47.99) | 3 (48-88) | *p* value | 1 (0-38.99) | 2 (39-49.99) | 3 (50-86.00) | *p* value |
| Mean age (SD) | | 42 (17.5) | 51 (18.2) | 55 (16.3) |  | 46 (18.0) | 49.8 (18.6) | 54 (17.6) |  |
| Sex (%) | Females | 28.05 | 35.35 | 36.60 | 0.001 | 26.69 | 35.24 | 38.07 | <0.001 |
| Race (%) | White | 31.47 | 34.99 | 33.54 | 0.015 | 37.90 | 32.74 | 29.36 | <0.001 |
|  | Black | 37.50 | 22.50 | 40.00 |  | 33.74 | 38.88 | 27.38 |  |
|  | Asian | 16.00 | 39.00 | 45.00 |  | 9.43 | 28.30 | 62.26 |  |
|  | Other | 35.14 | 24.32 | 40.54 |  | 29.68 | 34.30 | 36.01 |  |
| BMI, kg/m^2^ (%) | 18.5-24.9 | 26.07 | 31.80 | 42.46 | 0.035 | 28.50 | 32.58 | 38.92 | 0.107 |
|  | 25.0-29.9 | 29.43 | 35.13 | 35.44 |  | 25.84 | 34.86 | 39.30 |  |
|  | ≥30 | 37.09 | 37.09 | 26.03 |  | 36.45 | 34.06 | 29.49 |  |
| Education (%) | No Qual* | 35.65 | 35.65 | 28.44 | <0.001 | 35.69 | 34.90 | 29.41 | <0.001 |
|  | High School | 32.48 | 32.48 | 34.27 |  | 35.91 | 35.12 | 28.97 |  |
|  | Bachelors+ | 21.67 | 33.79 | 44.54 |  | 16.60 | 31.11 | 52.29 |  |
| Income Level (%) | Low^ | 35.41 | 39.06 | 25.54 | <0.001 | 36.92 | 34.33 | 28.75 | <0.001 |
|  | Middle^ | 31.74 | 32.12 | 36.14 |  | 34.68 | 33.86 | 31.47 |  |
|  | High^ | 25.18 | 33.15 | 41.67 |  | 24.69 | 33.63 | 41.67 |  |

U.K.=United Kingdom, U.S.=United States of America, U.K. “Other” group includes mixed race, U.S. “Other” group includes Hispanic, Mexican American and multiracial groups, *p* value based on Kruskal Wallis tests, age measured in years, SD=standard deviation, BMI (kg/m^2^) based on the WHO’s classification, No Qual*=no qualification,+=Bachelors degree and above, ^=NDNS categorised income into three tertiles, NHANES reported incomes were categorised into these tertiles based on distribution (low <$20,000, middle =$20,000-$64,999, high =$65,000+).

***Supplementary Table 3b:*** *Demographic characteristics by tertiles of DQI-I scores (U.K. vs. U.S.)*

|  | | **DQI-I (U.K.)** | | | | **DQI-I (U.S.)** | | | |
| --- | --- | --- | --- | --- | --- | --- | --- | --- | --- |
| Tertile (Score range) | | 1 (0-51) | 2 (52-62.99) | 3 (63-92) | *p* value | 1 (0-42) | 2 (43-54) | 3 (54-89) | *p* value |
| Mean age (SD) | | 46 (19.0) | 49 (17.7) | 53 (16.8) |  | 48 (18.9) | 49 (18.2) | 52 (17.8) |  |
| Sex (%) | Females | 35.98 | 34.02 | 30.01 | 0.368 | 34.49 | 31.85 | 33.66 | <0.001 |
| Race (%) | White | 33.49 | 32.74 | 33.78 | <0.001 | 35.73 | 37.07 | 35.73 | 0.009 |
|  | Black | 45.00 | 37.50 | 17.50 |  | 32.90 | 43.27 | 32.90 |  |
|  | Asian | 23.00 | 34.00 | 43.00 |  | 25.85 | 18.49 | 25.85 |  |
|  | Other | 32.43 | 48.65 | 18.92 |  | 34.47 | 25.24 | 34.47 |  |
| BMI, kg/m^2^ (%) | 18.5-24.9 | 32.62 | 32.46 | 35.25 | 0.186 | 29.92 | 31.53 | 38.54 | 0.067 |
|  | 25.0-29.9 | 31.28 | 32.97 | 35.75 |  | 27.68 | 34.86 | 37.46 |  |
|  | ≥30 | 31.24 | 35.36 | 33.62 |  | 38.62 | 33.62 | 27.76 |  |
| Education (%) | No Qual* | 40.58 | 31.16 | 28.26 | <0.001 | 34.77 | 29.67 | 35.42 | <0.001 |
|  | High School | 31.97 | 34.53 | 33.50 |  | 37.30 | 34.76 | 27.94 |  |
|  | Bachelors+ | 23.48 | 34.92 | 41.60 |  | 21.95 | 34.92 | 41.60 |  |
| Income Level (%) | Low | 44.85 | 30.26 | 24.89 | <0.001 | 39.92 | 29.84 | 30.25 | <0.001 |
|  | Medium | 30.21 | 32.70 | 37.09 |  | 34.38 | 34.73 | 30.88 |  |
|  | High | 22.10 | 37.32 | 40.58 |  | 29.07 | 33.18 | 37.75 |  |

U.K.=United Kingdom, U.S.=United States of America, U.K. “Other” group includes mixed race, U.S. “Other” group includes Hispanic, Mexican American and multiracial groups, *p* value based on Kruskal Wallis tests, age measured in years, SD=standard deviation, BMI (kg/m^2^) based on the WHO’s classification, No Qual*=no qualification, +=Bachelors degree and above, ^=NDNS categorised income into three tertiles, NHANES reported incomes were categorised into these tertiles based on distribution (low <$20,000, middle =$20,000-$64,999, high =$65,000+).

***Supplementary Table 3c:*** *Demographic characteristics by tertiles of EAT Lancet scores (U.K. vs. U.S.)*

|  | | **EAT-Lancet (U.K.)** | | | | **EAT-Lancet (U.S.)** | | | |
| --- | --- | --- | --- | --- | --- | --- | --- | --- | --- |
| Tertile (Score range) | | 1 (0-15.99) | 2 (16-18.99) | 3 (19-33) | *p* value | 1 (0-13.99) | 2 (14-17.99) | 3 (18-35) | *p* value |
| Mean age (SD) | | 47 (17.5) | 50 (19.3) | 51 (17.4) |  | 45 (18.1) | 50 (18.4) | 53 (17.5) |  |
| Sex (%) | Females | 28.50 | 26.18 | 45.33 | <0.001 | 26.38 | 35.60 | 38.02 | <0.001 |
| Race (%) | White | 33.54 | 26.40 | 40.06 | <0.001 | 32.36 | 38.22 | 29.43 | <0.001 |
|  | Black | 27.50 | 17.50 | 55.00 |  | 33.36 | 35.23 | 31.40 |  |
|  | Asian | 16.00 | 24.00 | 60.00 |  | 13.21 | 25.28 | 61.51 |  |
|  | Other | 21.62 | 27.03 | 51.35 |  | 26.35 | 36.18 | 37.47 |  |
| BMI, kg/m^2^ (%) | 18.5-24.9 | 29.51 | 23.11 | 47.70 | 0.002 | 25.38 | 33.33 | 41.29 | 0.235 |
|  | 25.0-29.9 | 30.82 | 26.66 | 42.53 |  | 26.45 | 34.79 | 38.76 |  |
|  | ≥30 | 39.05 | 27.33 | 33.84 |  | 32.10 | 37.21 | 30.69 |  |
| Income Level (%) | Low | 39.91 | 27.04 | 33.05 | <0.001 | 32.70 | 35.56 | 31.74 | <0.001 |
|  | Medium | 32.31 | 27.72 | 39.96 |  | 30.24 | 35.55 | 34.21 |  |
|  | High | 28.80 | 21.38 | 49.82 |  | 25.72 | 33.95 | 40.32 |  |
| Education (%) | No Qual* | 39.00 | 26.25 | 34.75 | <0.001 | 28.89 | 35.82 | 35.29 | <0.001 |
|  | High School | 30.18 | 31.20 | 38.62 |  | 32.46 | 36.51 | 31.03 |  |
|  | Bachelors+ | 21.95 | 34.92 | 41.60 |  | 19.37 | 32.16 | 48.47 |  |

U.K.=United Kingdom, U.S.=United States of America; U.K. “Other” group includes mixed race, U.S. “Other” group includes Hispanic, Mexican American and multiracial groups, *p* value based on Kruskal Wallis tests, age measured in years, SD=standard deviation, BMI (kg/m^2^) based on the WHO’s classification, No Qual*=no qualification, +=Bachelors degree and above, ^=NDNS categorised income into three tertiles, NHANES reported incomes were categorised into these tertiles based on distribution (low <$20,000, middle =$20,000-$64,999, high =$65,000+).
